# Prospective Observational Comparison of Demographic Characteristics, Vascular Risk Factors, Neurological Severity, and In-Hospital Outcomes Between Adult Patients With Acute Ischemic and Hemorrhagic Stroke

**DOI:** 10.7759/cureus.113822

**Published:** 2026-08-02

**Authors:** Syed Mohammad Hassan Raza Rizvi, Ahsan Amer, Ahmed Amer, Ayesha Siddiqua, Farzana Hakim, Ahsaan Amer

**Affiliations:** 1 Department of Surgery, Oslo Medical Center, Oslo, NOR; 2 Department of Medicine, Fauji Foundation Hospital, Rawalpindi, PAK; 3 Department of Geriatric Medicine, Royal Bournemouth Hospital, University Hospitals Dorset NHS Foundation Trust, Bournemouth, GBR; 4 Internal Medicine, MaxHealth Hospital Islamabad, Islamabad, PAK; 5 Department of Biochemistry, Foundation University Islamabad, Islamabad, PAK; 6 Medicine, Al-Nafees Medical College, Islamabad, PAK

**Keywords:** clinical characteristics, hemorrhagic stroke, ischemic stroke, risk factors, stroke outcomes

## Abstract

Background

Stroke is a leading cause of morbidity and mortality. Acute ischemic and hemorrhagic stroke differ in their demographic characteristics, vascular risk factor profiles, clinical presentation, neurological severity, and short-term outcomes. This study aimed to compare the demographic characteristics, vascular risk factors, clinical presentation, neurological severity, and in-hospital outcomes of adult patients presenting with acute ischemic and hemorrhagic stroke.

Methodology

This hospital-based, prospective, observational study was conducted at Fauji Foundation Hospital, Rawalpindi, from April 2024 to March 2025. A total of 240 adult patients with acute stroke presenting within 72 hours of symptom onset were enrolled. Stroke subtype was confirmed by neuroimaging using computed tomography and/or magnetic resonance imaging. Demographic characteristics, vascular risk factors, clinical features, neurological severity, and in-hospital outcomes were recorded using a standardized data collection proforma. Data were analyzed using SPSS version 26. Frequencies and percentages were calculated for categorical variables, while mean ± standard deviation was used for continuous variables. Normality of continuous variables was assessed before applying the independent-samples t-test. Comparisons between ischemic and hemorrhagic stroke groups were performed using the chi-square test and independent-samples t-test, as appropriate. A p-value <0.05 was considered statistically significant.

Results

Among the 240 patients included, 168 (70.0%) had ischemic stroke, and 72 (30.0%) had hemorrhagic stroke. Hypertension was more frequent among patients with hemorrhagic stroke (60/72, 83.3%) than ischemic stroke (120/168, 71.4%), whereas diabetes mellitus (88/168, 52.4% vs. 20/72, 27.8%) and dyslipidemia (70/168, 41.7% vs. 16/72, 22.2%) were more common among patients with ischemic stroke. Compared with ischemic stroke, patients with hemorrhagic stroke had significantly higher mean systolic blood pressure (178.8 ± 23.7 vs. 156.4 ± 18.3 mmHg), lower Glasgow Coma Scale scores (9.4 ± 3.1 vs. 13.0 ± 2.2), longer hospital stay (9.6 ± 4.1 vs. 7.0 ± 2.8 days), a higher proportion with severe neurological deficits (34/72, 47.2% vs. 32/168, 19.1%), and greater in-hospital mortality (12/72, 16.7% vs. 14/168, 8.3%). Chi-square and independent-samples t-tests demonstrated significant differences between ischemic and hemorrhagic stroke with respect to age, sex, hypertension, diabetes mellitus, dyslipidemia, blood pressure, Glasgow Coma Scale score, hospital stay, neurological severity, and in-hospital outcomes (all p < 0.05), whereas residence, smoking status, obesity, and previous stroke showed no significant between-group differences (p > 0.05).

Conclusions

Hemorrhagic stroke was associated with greater neurological severity, higher blood pressure, longer hospitalization, and poorer in-hospital outcomes than ischemic stroke, whereas diabetes mellitus and dyslipidemia were more frequently observed among patients with ischemic stroke. These findings reflect the relative frequency of selected risk factors within this study population and should not be interpreted as indicating that these factors are unimportant in the alternate stroke subtype or as a basis for differentiating stroke subtype in clinical practice. Prompt neuroimaging remains essential for accurate diagnosis and appropriate acute management.

## Introduction

One of the leading causes of mortality and permanent disability worldwide, stroke places a significant strain on health facilities, particularly in low- and middle-income nations [1]. It is a heterogeneous neurological disease that is manifested by sudden cessation of blood supply to the brain, leading to acute focal neurological deficits [2]. There are two main types of stroke: hemorrhagic stroke, which occurs when a brain artery enlarges or bursts, causing the brain to bleed, and acute ischemic stroke, which occurs when an artery is blocked by a clot or blood vessel rupture [3]. These two subtypes are very different in terms of their pathophysiology, clinical presentation, treatment strategy, and prognosis, and must be differentiated early in clinical practice with accuracy [4].

In South Asia, including Pakistan, the burden of stroke is rapidly rising, and the disease burden is caused by a growing prevalence of modifiable vascular risk factors, including hypertension, diabetes mellitus, dyslipidemia, smoking, obesity, and sedentary lifestyle [5,6]. Acute ischemic stroke is more common than hemorrhagic stroke, which, although less common, is associated with very high early mortality rates, rapid deterioration in neurological function, and poor short-term outcomes [7]. There may be variations in the distribution of stroke subtypes as well as in the clinical and demographic features of each due to variations in genetic susceptibility, risk factor profiles, and access to healthcare [8,9]. Consequently, evidence generated in other countries or healthcare systems may not be directly applicable to Pakistani patients, where disease patterns, healthcare access, referral pathways, and vascular risk factor profiles differ substantially.

In tertiary care centers, patients with stroke have a very broad spectrum of clinical presentations, from minor focal neurological deficits to impaired consciousness and life-threatening complications [10]. The presentation severity and the type of stroke are influenced by age, gender, comorbidities, and vascular risk factor burden. Knowledge of such patterns is crucial for improving early recognition, triage, and acute management of neurological emergencies [11,12]. Although several Pakistani studies have described the frequency or individual risk factors of stroke, most have focused on a single stroke subtype, specific risk factors, or isolated clinical outcomes. Few contemporary studies have comprehensively compared demographic characteristics, vascular risk factors, neurological severity, and short-term in-hospital outcomes between ischemic and hemorrhagic stroke within the same cohort using standardized clinical assessment.

Acute neurological emergencies constitute a major proportion of admissions to tertiary care hospitals in Rawalpindi, and stroke remains a substantial clinical burden. However, there is limited contemporary evidence from this region evaluating the full spectrum of demographic characteristics, vascular risk factors, neurological severity, and early hospital outcomes across stroke subtypes. Furthermore, regional differences in patient characteristics and healthcare delivery may influence stroke presentation and prognosis, highlighting the need for locally generated evidence to support clinical decision-making and resource allocation. By providing a comprehensive comparative evaluation of ischemic and hemorrhagic stroke in a large consecutive cohort of adult patients treated at a tertiary care hospital, the present study aims to address this knowledge gap and provide updated evidence relevant to stroke management in Pakistan. The objective of this research was to assess and compare the demographic characteristics, vascular risk factors, neurological severity, clinical presentation, and in-hospital outcomes of adult patients presenting with acute ischemic and hemorrhagic stroke.

## Materials and methods

Study design

A hospital-based, prospective, observational comparative study was conducted to evaluate and compare the demographic characteristics, vascular risk factors, clinical presentation, neurological severity, and in-hospital outcomes of adult patients with acute ischemic and hemorrhagic stroke.

Study setting

This study was conducted at Fauji Foundation Hospital, Rawalpindi, Department of Medicine and Neurology, a tertiary care facility managing a high volume of acute neurological emergencies.

Study duration

The study was conducted over a 12-month period from April 2024 to March 2025. Eligible patients were prospectively enrolled during this period to ensure adequate representation of seasonal variation and patient case flow.

Sample size and sampling technique

The sample size was calculated using the WHO sample size formula [13], assuming a prevalence of ischemic stroke of 66%, a 95% confidence level, and a 6% margin of error, which yielded a minimum required sample of 240 patients. Therefore, a total of 240 patients were included in the study.

$n = \frac{Z^{2} \times P \times (1-P)}{d^{2}}$

In the above formula, n is the required sample size, Z is the standard normal deviate at the 95% confidence level (1.96), P is the expected prevalence of ischemic stroke (66%) based on a previous Pakistani study [14], and d is the margin of error (6%). This calculation yielded a minimum sample size of approximately 240 participants. Because the primary objective of the study was to compare ischemic and hemorrhagic stroke, the adequacy of this sample size was further verified using the OpenEpi sample size calculator for comparison of two independent proportions (Fleiss method with continuity correction). The calculation was based on the prevalence of dyslipidemia reported by Bibi and Ali [15], in which dyslipidemia was present in 62.9% (44/70) of patients with ischemic stroke and 21.4% (15/70) of patients with hemorrhagic stroke. Assuming a 95% confidence level, 80% statistical power, and an equal allocation ratio, the calculated minimum sample size was substantially lower than the planned sample. Therefore, the enrollment of 240 consecutive patients exceeded the minimum requirement, providing adequate precision for both prevalence estimation and comparative analyses.

Non-probability consecutive sampling was employed. All adult patients presenting to Fauji Foundation Hospital, Rawalpindi during the study period with clinical features suggestive of acute stroke were screened for eligibility. Patients with a first admission for acute stroke who fulfilled the inclusion criteria and had radiologically confirmed ischemic or hemorrhagic stroke on computed tomography (CT) and/or magnetic resonance imaging (MRI) were consecutively enrolled until the required sample size was achieved. Recurrent admissions of the same patient during the study period were excluded to avoid duplicate observations. Consecutive recruitment of all eligible patients minimized selection bias and ensured representative inclusion of stroke cases encountered in routine clinical practice.

Inclusion and exclusion criteria

**Figure 1:**
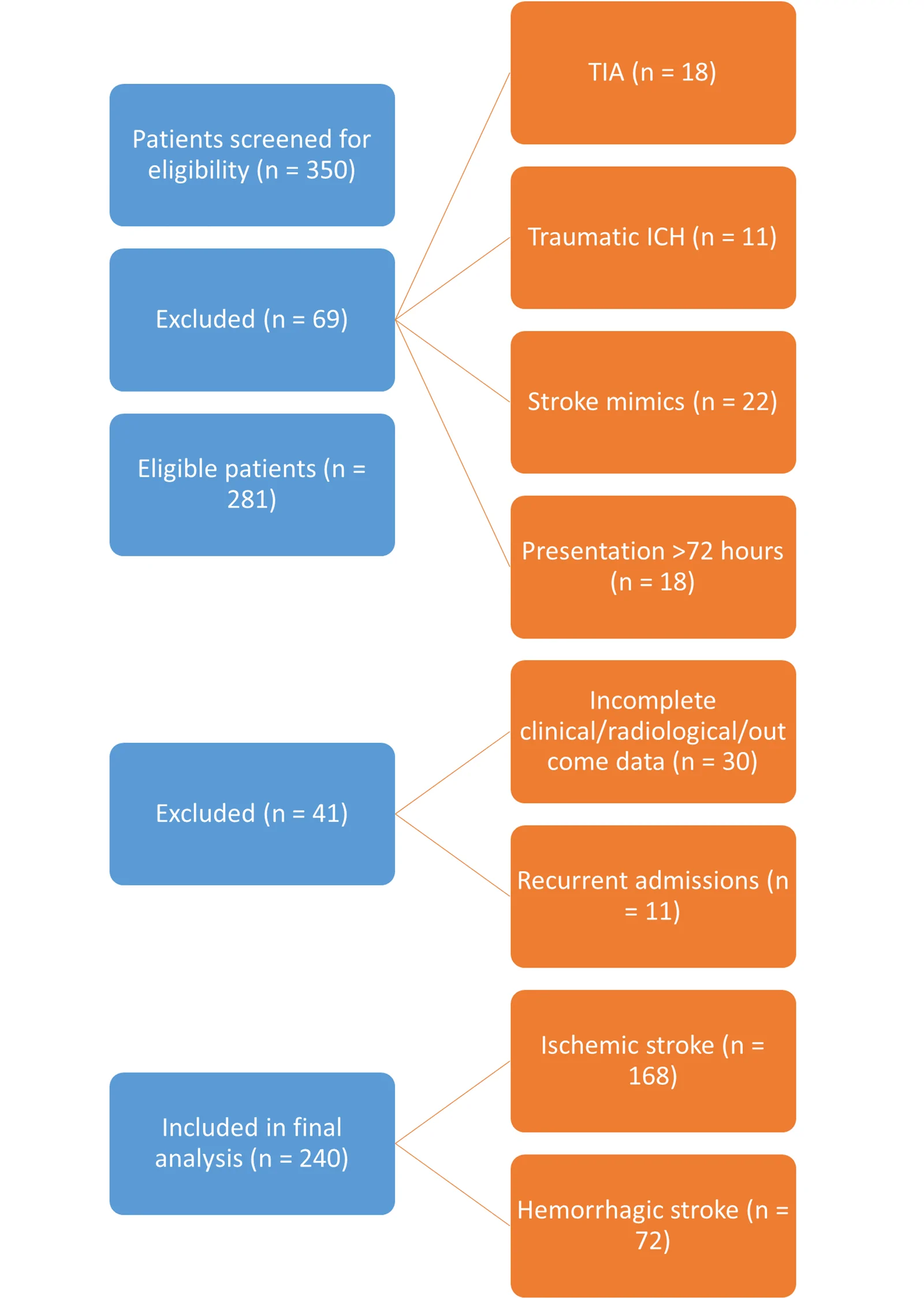
Flow diagram. TIA = transient ischemic attack; ICH = intracerebral hemorrhage

Included were patients aged 18 years or older presenting with acute neurological deficits suggestive of stroke and whose diagnosis of ischemic or hemorrhagic stroke was confirmed by CT and/or MRI. Eligible patients presented within 72 hours of symptom onset. Patients with transient ischemic attack, traumatic intracerebral hemorrhage, stroke mimics (including hypoglycemia, seizures, brain tumors, and metabolic encephalopathy), or insufficient clinical or radiological evidence were excluded. Only participants with complete demographic, clinical, laboratory, imaging, and in-hospital outcome data were included in the final analysis. Cases with missing key variables were excluded before statistical analysis. Recurrent admissions of the same patient during the study period were not analyzed separately, and only the first eligible admission was included to avoid duplication. The participant flow diagram is shown in Figure 1.

Data collection procedure

A standardized structured data collection proforma was specifically developed by the study investigators after an extensive review of the published literature and established stroke registries to ensure comprehensive capture of demographic characteristics, vascular risk factors, clinical presentation, neurological findings, investigations, treatment, and in-hospital outcomes. The proforma underwent face and content validity assessment by a panel comprising two consultant neurologists, one consultant physician, and one epidemiologist, who evaluated its relevance, clarity, completeness, and clinical applicability. Necessary modifications were incorporated based on their recommendations before commencement of the study.

Before patient recruitment, the proforma was pilot tested in 15 consecutive patients with acute stroke who were not included in the final analysis to evaluate feasibility, clarity, completeness, and data consistency. Minor wording and formatting revisions were made following pilot testing to improve usability without altering the study variables.

All eligible patients were prospectively assessed in the Emergency Department and Neurology wards of Fauji Foundation Hospital, Rawalpindi. Stroke subtype was confirmed by CT and/or MRI brain imaging. Clinical, demographic, laboratory, treatment, and outcome data were collected prospectively from hospital admission until discharge using the standardized study proforma. Smoking history was obtained through direct patient interview or, when necessary, from an accompanying family member using the standardized study proforma. Only cigarette smoking was recorded, whereas smokeless tobacco products and electronic nicotine delivery systems were not assessed. Data collection was performed by trained physicians under the supervision of consultant neurologists to ensure consistency and minimize observer variability.

Neurological severity was assessed at the time of initial clinical evaluation using patients’ level of consciousness, motor deficits, and overall functional impairment, and was categorized into mild, moderate, or severe according to a predefined study-specific clinical classification for descriptive stratification. This pragmatic classification was developed to facilitate bedside assessment within the local clinical setting and was not intended to replace internationally validated stroke severity instruments. To provide an objective and standardized assessment of neurological status, the Glasgow Coma Scale (GCS) score was recorded for all participants at presentation and analyzed as a continuous variable in both univariate and multivariable analyses. Although validated stroke-specific instruments were not routinely collected during the study period, incorporation of the GCS score provided an objective measure of neurological impairment and enabled adjustment for neurological severity when evaluating in-hospital outcomes.

Study variables

The primary exposure variable was stroke subtype (ischemic or hemorrhagic stroke). Independent variables included demographic characteristics (age, sex, and residence), vascular risk factors (hypertension, diabetes mellitus, dyslipidemia, smoking status, obesity, and previous stroke), and clinical parameters recorded at presentation, including systolic blood pressure, diastolic blood pressure, GCS score, neurological severity category, and hospital length of stay. The primary outcome for multivariable analysis was poor in-hospital outcome, defined as the composite of intensive care unit (ICU) admission or in-hospital mortality. Secondary outcomes included neurological severity, duration of hospitalization, ICU admission, and in-hospital mortality.

Operational definitions

Ischemic Stroke

Acute onset of focal or global neurological deficit caused by cerebral infarction, confirmed by CT and/or MRI.

Hemorrhagic Stroke

Acute onset of focal or global neurological deficit caused by spontaneous intracerebral hemorrhage, confirmed by CT and/or MRI.

Hypertension

Systolic blood pressure ≥140 mmHg and/or diastolic blood pressure ≥90 mmHg, or current use of antihypertensive medication.

Diabetes Mellitus

Previous physician-diagnosed diabetes mellitus, current use of antidiabetic medication, or documented diagnosis in the medical record.

Dyslipidemia

Previous physician-diagnosed dyslipidemia, current use of lipid-lowering therapy, or an abnormal lipid profile according to institutional laboratory reference ranges.

Neurological Severity

Neurological impairment at presentation was classified as mild, moderate, or severe according to a predefined study-specific clinical classification based on level of consciousness, motor deficit, and functional impairment. This classification was developed to facilitate descriptive clinical stratification within the study setting and has not undergone formal external validation. The GCS score was additionally recorded for all patients as an objective measure of neurological status and analyzed as a continuous variable.

Mild neurological severity: Patients with preserved consciousness, mild focal neurological deficits, and minimal functional impairment without major limitation of daily activities.

Moderate neurological severity: Patients with moderate impairment of consciousness and/or motor function resulting in clinically significant neurological deficits requiring hospital-based management but without profound neurological dysfunction.

Severe neurological severity: Patients with marked impairment of consciousness, severe motor deficits, or extensive neurological dysfunction associated with substantial functional impairment and a high likelihood of intensive medical management.

Smoking

Smoking status was determined by self-report or information provided by an accompanying family member. Participants were classified as smokers if they had a history of regular cigarette smoking (current or former). Smokeless tobacco products (e.g., naswar, gutka, paan containing tobacco) and electronic cigarettes were not included.

Improved/Stable Outcome

Patient discharged alive without neurological deterioration during hospitalization, with either clinical improvement or maintenance of neurological status without further decline.

Intensive Care Unit Admission

Transfer to the ICU during the index hospitalization because of neurological deterioration or the need for intensive monitoring or advanced supportive care.

In-Hospital Mortality

Death from any cause occurring during the index hospitalization before discharge.

Statistical analysis

The data were analyzed using SPSS version 26.0 (IBM Corp., Armonk, NY, USA). Continuous variables were summarized as mean ± standard deviation (SD), while categorical variables were presented as frequencies and percentages. The normality of continuous variables was assessed using the Shapiro-Wilk test. Between-group comparisons of continuous variables were performed using the independent-samples t-test, whereas categorical variables were compared using the chi-square test. The Mann-Whitney U test was prespecified for non-normally distributed continuous variables; however, it was not required because all analyzed continuous variables satisfied the assumption of normality.

To account for potential confounding and identify independent predictors of poor in-hospital outcome, a multivariable binary logistic regression analysis was performed. The dependent variable was poor in-hospital outcome, defined as a composite endpoint of ICU admission or in-hospital mortality. Independent variables entered into the model included stroke subtype (hemorrhagic vs. ischemic, with ischemic stroke as the reference category), age (continuous), sex (male vs. female), hypertension, diabetes mellitus, dyslipidemia, systolic blood pressure (continuous), and GCS score (continuous). These covariates were selected a priori based on their established clinical relevance and statistically significant or clinically important associations identified in the univariate analyses. Model performance was evaluated using the likelihood ratio test and Nagelkerke’s pseudo R², and results were reported as adjusted odds ratios (aORs) with 95% confidence intervals (CIs). All statistical tests were two-tailed, and a p-value <0.05 was considered statistically significant.

Ethical considerations

Ethical approval was obtained from the Institutional Review Board of Fauji Foundation Hospital, Rawalpindi (approval number: 1218/DM/FJHR). In accordance with the standards for ethical conduct in research, all patients or their attendants provided written informed permission, and patient data confidentiality was maintained throughout the study.

## Results

**Figure 2:**
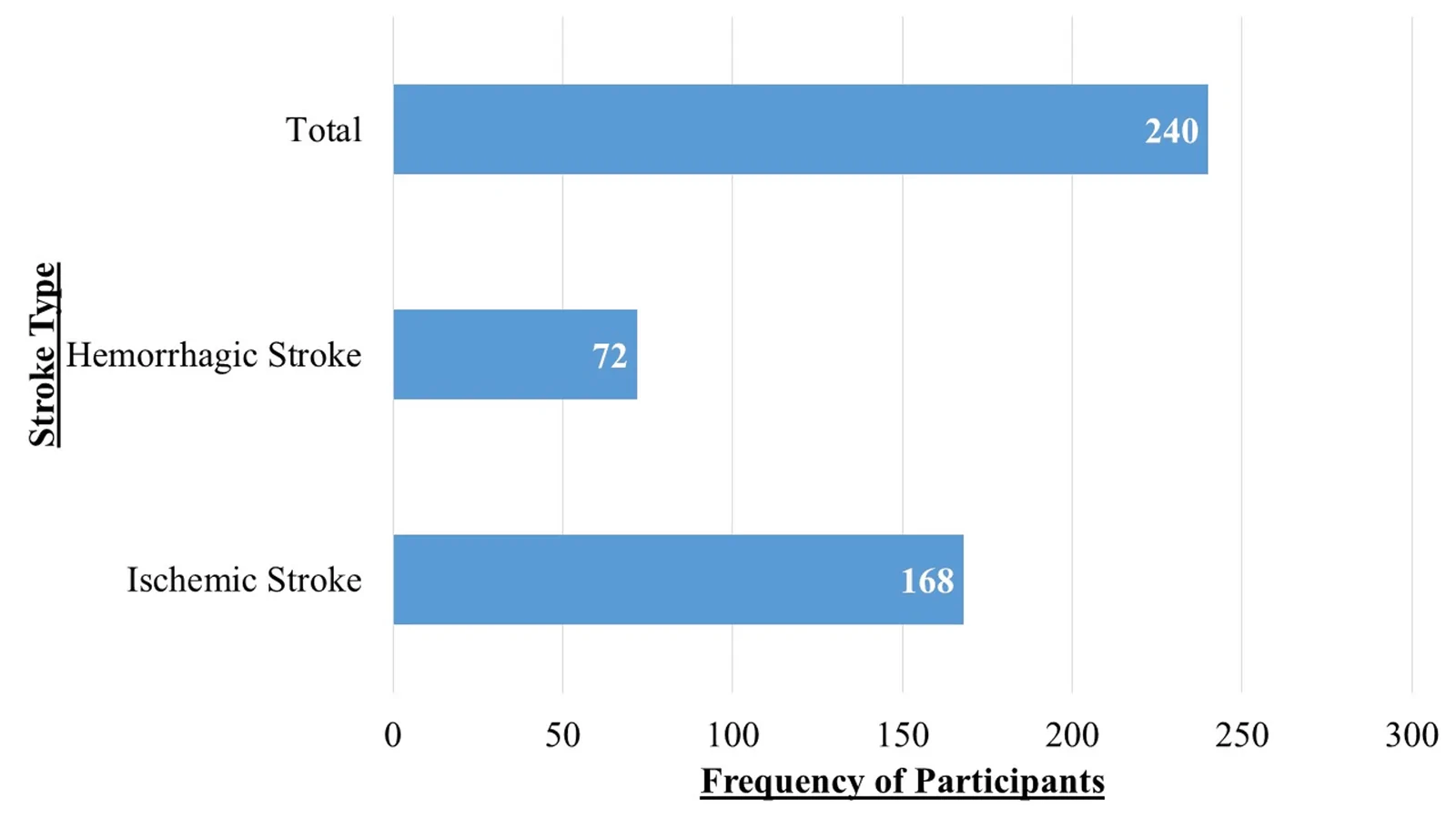
Distribution of patients according to stroke type (n = 240).

Figure 2 illustrates the distribution of stroke subtypes in the study population. Ischemic stroke was the predominant type, accounting for the majority of cases, indicating a higher burden of ischemic cerebrovascular events compared to hemorrhagic stroke in the studied cohort.

Table 1 summarizes the baseline characteristics of 240 stroke patients (168 ischemic, 72 hemorrhagic). Ischemic stroke patients were significantly older and more likely to be female, whereas hemorrhagic stroke occurred more frequently in younger males. The ischemic group had a higher burden of diabetes mellitus and dyslipidemia, while hypertension was more prevalent among hemorrhagic stroke patients. Hemorrhagic stroke was associated with markedly elevated blood pressure, lower GCS scores, greater neurological severity, and significantly poorer in-hospital outcomes, including higher ICU admission and mortality rates. Smoking, obesity, previous stroke history, and place of residence did not differ significantly between subtypes.

**Table 1:** Baseline demographic characteristics and vascular risk factors. Data are presented as mean ± SD for continuous variables and as n (%) for categorical variables. Continuous variables were compared between groups using the independent samples t-test. Categorical variables were compared using the chi-square (χ²) test. *: Statistically significant at p < 0.05.

Variable	Category	Ischemic (n = 168)	Hemorrhagic (n = 72)	Statistic	P-value*
Age (years)	Mean ± SD	62.14 ± 10.86	57.28 ± 11.94	t = 2.59	0.010
	18–40	22 (13.1)	18 (25.0)	χ² = 7.13	0.028
	41–60	74 (44.0)	30 (41.7)		
	>60	72 (42.9)	24 (33.3)		
Gender	Male	104 (61.9)	50 (69.4)	χ² = 4.17	0.041
	Female	64 (38.1)	22 (30.6)		
Residence	Urban	102 (60.7)	38 (52.8)	χ² = 2.44	0.118
	Rural	66 (39.3)	34 (47.2)		
Hypertension	Yes	120 (71.4)	60 (83.3)	χ² = 3.91	0.048
	No	48 (28.6)	12 (16.7)		
Diabetes mellitus	Yes	88 (52.4)	20 (27.8)	χ² = 11.72	0.001
	No	80 (47.6)	52 (72.2)		
Smoking	Yes	76 (45.2)	40 (55.6)	χ² = 2.14	0.146
	No	92 (54.8)	32 (44.4)		
Dyslipidemia	Yes	70 (41.7)	16 (22.2)	χ² = 7.60	0.006
	No	98 (58.3)	56 (77.8)		
Obesity	Yes	54 (32.1)	18 (25.0)	χ² = 1.21	0.276
	No	114 (67.9)	54 (75.0)		
Previous stroke	Yes	38 (22.6)	10 (13.9)	χ² = 2.29	0.129
	No	130 (77.4)	62 (86.1)		

Table 2 compares mean clinical parameters between ischemic and hemorrhagic stroke patients. Hemorrhagic stroke was associated with significantly higher blood pressure values and lower GCS scores, reflecting greater acute physiological derangement. In contrast, ischemic stroke patients demonstrated a comparatively better level of consciousness and shorter hospital stay. Overall, the results indicate more severe clinical presentation among hemorrhagic stroke cases.

**Table 2:** Clinical presentation and neurological severity. Continuous variables are expressed as mean ± SD and were compared using the independent-samples t-test. Neurological severity categories are presented as frequency (n) and percentage (%) and were compared using the chi-square (χ²) test. Neurological severity was categorized at presentation as mild, moderate, or severe according to the predefined study-specific clinical classification. BP = blood pressure; GCS = Glasgow Coma Scale; SD = standard deviation

Variable		Ischemic	Hemorrhagic	Statistic	P-value
Clinical measurements	Systolic BP	156.42 ± 18.26	178.84 ± 23.68	t = 7.41	<0.001
	Diastolic BP	94.18 ± 11.24	106.22 ± 13.96	t = 6.52	<0.001
	GCS score	13.02 ± 2.18	9.44 ± 3.08	t = 9.21	<0.001
	Hospital stay (days)	6.96 ± 2.84	9.62 ± 4.06	t = 3.19	0.002
Neurological severity	Mild	54 (32.1)	10 (13.9)	χ² = 12.31	0.002
	Moderate	82 (48.8)	28 (38.9)		
	Severe	32 (19.1)	34 (47.2)		

Table 3 shows the distribution of neurological severity according to stroke subtype. Hemorrhagic stroke was more frequently associated with severe neurological impairment, whereas ischemic stroke cases were predominantly categorized as mild to moderate. The observed differences indicate a significantly higher neurological burden in hemorrhagic stroke patients compared to ischemic stroke.

**Table 3:** Treatment characteristics, in-hospital outcomes, and multivariable predictors of poor in-hospital outcome according to stroke subtype (n = 240). Data are presented as frequency (n) and percentage (%) unless otherwise indicated. Categorical variables were compared using the Chi-square (χ²) test or Fisher’s exact test, as appropriate. Multivariable binary logistic regression was performed to identify independent predictors of poor in-hospital outcome, defined as a composite of ICU admission or in-hospital mortality. aORs with 95% CIs are presented. The multivariable model included stroke subtype, age, sex, hypertension, diabetes mellitus, dyslipidemia, systolic blood pressure, and GCS score. Model fit was assessed using the likelihood ratio test and Nagelkerke’s pseudo-R². A two-sided p < 0.05 was considered statistically significant. aOR = adjusted odds ratio; BP = blood pressure; CI = confidence interval; GCS = Glasgow Coma Scale; ICU = intensive care unit

Section	Variable	Ischemic stroke (n = 168)	Hemorrhagic stroke (n = 72)	Univariate test	aOR (95% CI)	P-value
Treatment characteristics	Thrombolysis, n (%)	22 (13.10)	0 (0.00)	Fisher’s exact <0.001	—	—
	ICU admission, n (%)	10 (5.95)	18 (25.00)	χ² = 18.64	—	<0.001
In-hospital outcomes	Improved/Stable, n (%)	144 (85.71)	42 (58.33)	χ² = 18.64	—	<0.001
	In-hospital mortality, n (%)	14 (8.33)	12 (16.67)	χ² = 4.94	—	0.026*
Multivariable logistic regression	Hemorrhagic stroke (vs. ischemic)	—	—	—	2.75 (0.99–7.67)	0.053
	Age (per year)	—	—	—	1.00 (0.94–1.05)	0.896
	Male sex	—	—	—	0.37 (0.10–1.44)	0.154
	Hypertension	—	—	—	2.98 (0.87–10.21)	0.082
	Diabetes mellitus	—	—	—	3.61 (0.93–14.01)	0.063
	Dyslipidemia	—	—	—	0.48 (0.15–1.53)	0.213
	Systolic BP (per mmHg)	—	—	—	1.00 (0.99–1.02)	0.736
	GCS score (per point)	—	—	—	0.80 (0.69–0.94)	0.005

A multivariable binary logistic regression analysis was performed to identify independent predictors of poor in-hospital outcome, defined as the composite of ICU admission or in-hospital mortality (Table 4). The model included stroke subtype, age, sex, hypertension, diabetes mellitus, dyslipidemia, systolic blood pressure, and GCS score as covariates, with results reported as aORs and 95% CIs. After adjustment for potential confounders, GCS score was the only independent predictor of poor in-hospital outcome, with each 1-point increase in GCS associated with a 20% reduction in the odds of poor outcome (aOR = 0.80, 95% CI: 0.69-0.94, p = 0.005). Although hemorrhagic stroke demonstrated a higher risk of poor outcome on univariate analysis, it did not remain statistically significant after adjustment (aOR = 2.75, 95% CI: 0.99-7.67, p = 0.053), suggesting that its association with adverse outcomes was largely explained by greater neurological impairment and acute physiological derangement at presentation. Hypertension (p = 0.082) and diabetes mellitus (p = 0.063) showed borderline associations with poor outcome, whereas age, sex, dyslipidemia, and systolic blood pressure were not independently associated. The overall model demonstrated acceptable performance (likelihood ratio χ² = 37.62, Nagelkerke pseudo-R² = 0.147).

**Table 4:** Multivariate logistic regression (poor in-hospital outcome). Model fit: Pseudo R² = 0.147; LR χ² = 37.62, *: p < 0.05 is significant. aOR = adjusted odds ratio; CI = confidence interval; GCS = Glasgow Coma Scale; ICU = intensive care unit; LR = likelihood ratio; BP = blood pressure

Variable	Adjusted OR	95% CI (lower–upper)	P-value
Hemorrhagic stroke (vs. ischemic)	2.75	0.99–7.67	0.053
Age (per year)	1	0.94–1.05	0.896
Male gender (vs. female)	0.37	0.10–1.44	0.154
Hypertension	2.98	0.87–10.21	0.082
Diabetes mellitus	3.61	0.93–14.01	0.063
Dyslipidemia	0.48	0.15–1.53	0.213
Systolic BP (per mmHg)	1	0.99–1.02	0.736
GCS score (per point)	0.80	0.69–0.94	0.005*

## Discussion

In the present study, 240 patients with acute stroke were evaluated, of whom 168 (70.00%) had ischemic stroke and 72 (30.00%) had hemorrhagic stroke. This predominance of ischemic stroke is consistent with global epidemiological trends, where ischemic events account for approximately two-thirds to three-quarters of all strokes. Similarly, a tertiary care study from Pakistan reported that ischemic stroke constituted 66% of cases, while hemorrhagic stroke accounted for 34%, supporting the predominance of ischemic stroke in South Asian populations [14]. The higher proportion of ischemic stroke is biologically plausible because progressive atherosclerosis, endothelial dysfunction, diabetes mellitus, and dyslipidemia promote thromboembolic arterial occlusion more frequently than spontaneous intracerebral hemorrhage. Furthermore, improved neuroimaging availability has enhanced early recognition of ischemic stroke, whereas primary prevention strategies for vascular risk factors remain suboptimal in many low- and middle-income countries. These findings emphasize the importance of aggressive cardiovascular risk factor modification through hypertension control, diabetes management, lipid-lowering therapy, smoking cessation, and public awareness programs to reduce the future burden of ischemic stroke.

From the age perspective, patients aged 41-60 years constituted the largest proportion of ischemic stroke, whereas hemorrhagic stroke occurred relatively more frequently among older patients. Age was significantly associated with stroke subtype (p = 0.028). Similar findings have been reported by the Aga Khan University Stroke Registry, where the mean age of stroke patients was approximately 60 years [16]. The influence of age is biologically explained by cumulative vascular injury, prolonged exposure to hypertension, progressive arterial stiffness, chronic endothelial dysfunction, and increasing atherosclerotic burden over time. In addition, age-related cerebral small-vessel disease predisposes elderly individuals to both ischemic infarction and intracerebral hemorrhage. From a public health perspective, these findings highlight the need for earlier cardiovascular risk assessment and preventive interventions during middle adulthood rather than waiting until age, particularly in resource-limited healthcare systems such as Pakistan.

Male predominance was observed in both ischemic and hemorrhagic stroke, although the difference between stroke subtypes was not statistically significant (p = 0.335). Similar findings have been reported in Pakistani studies, where males consistently account for the majority of hospitalized stroke patients, reflecting the higher burden of vascular risk factors among men in the region [17,18]. Aziz et al. likewise observed a predominance of male patients with stroke in a tertiary care setting [17]. This pattern is likely attributable to greater exposure of men to modifiable vascular risk factors such as tobacco use, occupational stress, uncontrolled hypertension, and other lifestyle-related behaviors. Furthermore, delayed symptom recognition, sociocultural barriers, and disparities in healthcare access among women may contribute to their underrepresentation in hospital-based cohorts. Collectively, these findings emphasize the importance of sex-specific preventive strategies and equitable access to acute stroke services.

Regarding vascular risk factors, hypertension was significantly more prevalent among patients with hemorrhagic stroke than ischemic stroke, whereas diabetes mellitus and dyslipidemia were more common among ischemic stroke patients. Comparable observations have been reported by Bibi and Ali [15] and Aziz et al. [17], who demonstrated hypertension as the dominant risk factor for hemorrhagic stroke, whereas metabolic disorders were more strongly associated with ischemic stroke [15,17]. These findings are biologically plausible because chronic uncontrolled hypertension induces lipohyalinosis, fibrinoid necrosis, and structural weakening of small penetrating cerebral arteries, predisposing them to rupture and intracerebral hemorrhage. Conversely, diabetes mellitus promotes endothelial dysfunction, chronic inflammation, oxidative stress, and accelerated atherosclerosis, while dyslipidemia contributes to plaque formation and arterial occlusion, increasing the likelihood of ischemic stroke. Therefore, aggressive blood pressure control remains the cornerstone of hemorrhagic stroke prevention, whereas optimization of glycemic status and lipid levels is essential for reducing ischemic stroke risk. These findings further support strengthening community-based cardiovascular risk screening and long-term management programs in Pakistan.

Patients with hemorrhagic stroke demonstrated significantly higher systolic and diastolic blood pressure, lower GCS scores, and more severe neurological impairment than patients with ischemic stroke. Similar differences in neurological severity have been described in Pakistani cohorts by Khealani et al. and by Younis et al., who reported poorer neurological status and higher severity scores among patients with hemorrhagic stroke [18,19]. These findings are biologically plausible because intracerebral hemorrhage produces an expanding hematoma, perihematomal edema, mass effect, increased intracranial pressure, and secondary neuronal injury, leading to rapid deterioration in consciousness and neurological function. In contrast, ischemic stroke generally causes localized cerebral ischemia without the immediate space-occupying effects associated with intracranial bleeding. From a clinical perspective, these differences highlight the need for rapid neuroimaging, early blood pressure optimization, timely neurosurgical consultation, and prompt transfer of patients with suspected hemorrhagic stroke to intensive care facilities whenever indicated.

Analysis of in-hospital outcomes demonstrated significantly poorer prognosis among patients with hemorrhagic stroke, including higher ICU admission rates, longer hospital stay, higher rates of poor outcome, and increased in-hospital mortality. These findings are consistent with recent Pakistani studies by Bibi and Ali and Younis et al., both of which demonstrated significantly higher mortality and poorer functional outcomes among patients with hemorrhagic stroke [15,19]. International evidence from the Global Burden of Disease Stroke Collaborators and the INTERSTROKE investigators has similarly shown that intracerebral hemorrhage carries substantially greater early mortality and disability than ischemic stroke because of rapid hematoma expansion, elevated intracranial pressure, secondary brain injury, limited acute therapeutic options, and frequent need for neurosurgical intervention and intensive care [20,21]. In our multivariable logistic regression analysis, lower GCS score remained the strongest independent predictor of poor in-hospital outcome after adjustment for stroke subtype and major clinical confounders, indicating that neurological impairment at presentation is a more powerful determinant of early prognosis than stroke subtype alone. From a healthcare system perspective, these findings emphasize the need to establish dedicated stroke units, improve access to emergency neuroimaging, optimize critical care resources, and implement standardized evidence-based stroke pathways to improve survival and functional recovery among patients with acute stroke.

The observed differences in vascular risk factor frequencies should be interpreted with caution. Although hypertension was more frequently observed among patients with hemorrhagic stroke, while diabetes mellitus and dyslipidemia were more common among patients with ischemic stroke, these findings indicate only the relative distribution of selected risk factors within our study cohort. They should not be interpreted as evidence that these factors are unimportant contributors to the alternate stroke subtype. Considerable overlap exists in the vascular risk profiles of ischemic and hemorrhagic stroke, and many established risk factors contribute to both conditions. Moreover, the present study evaluated only a limited number of conventional vascular risk factors and did not include several recognized or emerging determinants of stroke, such as illicit drug use (particularly amphetamine or methamphetamine use), electronic cigarette (vaping) exposure, environmental factors, genetic predisposition, inflammatory biomarkers, dietary factors, psychosocial stressors, and other lifestyle-related risk factors. Consequently, these findings should not be interpreted as representing the complete spectrum of stroke risk factors or as a basis for distinguishing stroke subtype in clinical practice. Because clinical features and vascular risk factors substantially overlap between ischemic and hemorrhagic stroke, urgent neuroimaging with CT and/or MRI remains indispensable for accurate diagnosis and timely initiation of appropriate evidence-based treatment.

Study strengths and limitations

The present study has several important strengths. It employed a prospective observational comparative design with consecutive enrollment of 240 adult patients, thereby minimizing selection bias and ensuring systematic data collection from hospital admission until discharge. Stroke subtype was confirmed using CT and/or MRI, enhancing diagnostic accuracy and reducing disease misclassification. Clinical information was collected using a standardized study proforma that underwent expert review, pilot testing, and uniform implementation by trained investigators, improving the consistency and reproducibility of data collection. To further minimize measurement and information bias, standardized operational definitions were applied for all study variables, data were collected prospectively using predefined protocols, duplicate admissions were excluded, and only the first eligible admission for each patient was analyzed. Patients with incomplete key clinical or outcome variables were excluded before statistical analysis to ensure data completeness. Furthermore, a participant flow diagram was included to improve transparency regarding patient screening, eligibility assessment, exclusions, and final enrollment. Unlike many previous regional studies that evaluated isolated clinical characteristics or focused on a single stroke subtype, the present study provided a comprehensive comparison of demographic characteristics, vascular risk factors, clinical presentation, neurological severity, treatment characteristics, and short-term in-hospital outcomes within the same cohort. Neurological status was objectively assessed using the GCS, an internationally validated neurological assessment tool, and multivariable binary logistic regression was performed to adjust for clinically relevant confounding variables and identify independent predictors of poor in-hospital outcome. Collectively, these methodological measures reduced the risk of selection, measurement, and confounding bias, thereby strengthening the internal validity and clinical relevance of the study findings.

Despite these strengths, several limitations should be considered when interpreting the results. First, as a single-center, hospital-based, prospective, observational study, the findings may not be fully generalizable to the broader Pakistani population or to healthcare systems with different referral patterns, patient characteristics, and clinical practices. Second, although multivariable analysis was performed to adjust for major confounding variables, the observational nature of the study precludes causal inference, and residual or unmeasured confounding cannot be completely excluded. Third, long-term follow-up was not undertaken; therefore, important outcomes such as functional recovery, recurrent stroke, long-term disability, quality of life, and post-discharge mortality could not be evaluated, limiting assessment of long-term treatment effectiveness and outcome heterogeneity. Fourth, although GCS was recorded for all participants and incorporated into both univariate and multivariable analyses as an objective standardized neurological assessment, the absence of the National Institutes of Health Stroke Scale and modified Rankin Scale may limit direct comparison with international studies. Fifth, the study evaluated only selected conventional vascular risk factors and did not assess several established and emerging stroke risk factors, including illicit drug use (particularly amphetamine or methamphetamine use), electronic cigarette (vaping) exposure, genetic susceptibility, inflammatory biomarkers, dietary and environmental factors, and other lifestyle-related determinants. Consequently, the absence of these variables should not be interpreted as evidence that they are unimportant contributors to either ischemic or hemorrhagic stroke. Likewise, the observed differences in the frequency of selected risk factors between stroke subtypes should not be interpreted as sufficient for clinical differentiation of ischemic and hemorrhagic stroke. Given the substantial overlap in stroke risk factors, definitive classification continues to rely on prompt neuroimaging using CT and/or MRI.

Future prospective

Future multicenter prospective cohort studies with larger, nationally representative sample sizes, standardized stroke assessment using internationally validated instruments such as the National Institutes of Health Stroke Scale, modified Rankin Scale, Barthel Index, and Glasgow Outcome Scale,comprehensive treatment data, and extended longitudinal follow-up incorporating a broader range of conventional and emerging stroke risk factors, including substance use, electronic cigarette (vaping) exposure, genetic factors, inflammatory biomarkers, and environmental determinants are warranted to validate these findings, evaluate treatment effect heterogeneity, develop robust risk stratification models, and improve evidence-based stroke management and long-term patient outcomes in Pakistan.

## Conclusions

This prospective observational study demonstrates important differences between acute ischemic and hemorrhagic stroke with respect to demographic characteristics, vascular risk factor profiles, neurological severity, and early in-hospital outcomes. Hemorrhagic stroke was associated with more severe neurological impairment, markedly elevated blood pressure, longer hospitalization, higher rates of ICU admission, and greater in-hospital mortality, whereas diabetes mellitus and dyslipidemia were more frequently observed among patients with ischemic stroke. After adjustment for major demographic and clinical confounders, lower GCS score emerged as the strongest independent predictor of poor in-hospital outcome, emphasizing the prognostic importance of neurological status at presentation. These findings should be interpreted as comparative observations of the relative frequency of selected risk factors rather than evidence that particular risk factors are exclusive to one stroke subtype or unimportant in the other. Furthermore, the evaluated variables do not encompass the full spectrum of established or emerging stroke risk factors. Consequently, the results should not be used to distinguish ischemic from hemorrhagic stroke in clinical practice. Because substantial overlap exists in clinical presentation and vascular risk factors, rapid neuroimaging remains the cornerstone for accurate stroke subtype diagnosis and selection of appropriate acute treatment. These findings highlight the need for early recognition of stroke subtype, aggressive management of modifiable vascular risk factors, prompt neurological assessment, and timely implementation of evidence-based acute stroke care pathways to improve patient outcomes. Future multicenter prospective studies incorporating validated neurological assessment scales and long-term functional follow-up are warranted to further refine risk stratification and optimize stroke management strategies in Pakistan.

## Appendices

**Table 5:** Data collection questionnaire.

Section	Variable	Description/Options	Response
A. Administrative information			
A1	Study ID		
A2	Date of admission		
A3	Date of enrollment		
A4	Hospital registration number		
A5	Ward	Emergency=1, Neurology=2, ICU=3	
B. Demographic characteristics			
B1	Age (years)	Actual completed years	
B2	Age category	18–40=1, 41–60=2, >60=3	
B3	Gender	Male=1, Female=2	
B4	Residence	Urban=1, Rural=2	
C. Stroke characteristics			
C1	Stroke type	Ischemic=1, Hemorrhagic=2	
C2	CT brain performed	Yes=1, No=0	
C3	MRI brain performed	Yes=1, No=0	
C4	Radiological confirmation	CT=1, MRI=2, Both=3	
C5	Time since symptom onset	Hours	
C6	Presented within 72 hours	Yes=1, No=0	
D. Clinical presentation			
D1	Level of consciousness	Alert=1, Drowsy=2, Unconscious=3	
D2	Glasgow Coma Scale (GCS)	Score (3–15)	
D3	Neurological severity	Mild=1, Moderate=2, Severe=3	
D4	Motor weakness	Yes=1, No=0	
D5	Slurred speech	Yes=1, No=0	
D6	Aphasia	Yes=1, No=0	
D7	Facial weakness	Yes=1, No=0	
D8	Headache	Yes=1, No=0	
D9	Vomiting	Yes=1, No=0	
D10	Seizures	Yes=1, No=0	
D11	Visual disturbance	Yes=1, No=0	
D12	Sensory deficit	Yes=1, No=0	
D13	Limb weakness	Right=1, Left=2, Bilateral=3, None=0	
E. Vital signs			
E1	Systolic blood pressure	mmHg	
E2	Diastolic blood pressure	mmHg	
E3	Pulse rate	beats/min	
E4	Respiratory rate	breaths/min	
E5	Body temperature	°C	
F. Vascular risk factors			
F1	Hypertension	Yes=1, No=0	
F2	Diabetes mellitus	Yes=1, No=0	
F3	Dyslipidemia	Yes=1, No=0	
F4	Smoking status	Current=1, Former=2, Never=3	
F5	Obesity	Yes=1, No=0	
F6	Previous stroke	Yes=1, No=0	
F7	Family history of stroke	Yes=1, No=0	
G. Laboratory findings			
G1	Random blood glucose	mg/dL	
G2	Hemoglobin	g/dL	
G3	Serum creatinine	mg/dL	
G4	Lipid profile available	Yes=1, No=0	
H. Hospital management			
H1	Thrombolysis received	Yes=1, No=0	
H2	Neurosurgical intervention	Yes=1, No=0	
H3	ICU admission	Yes=1, No=0	
H4	Mechanical ventilation	Yes=1, No=0	
H5	Hospital-acquired pneumonia	Yes=1, No=0	
I. Hospital course			
I1	Length of hospital stay	Days	
I2	Clinical deterioration	Yes=1, No=0	
I3	Referred to ICU during admission	Yes=1, No=0	
J. In-hospital outcomes			
J1	Outcome status	Improved=1, Stable=2, Poor Outcome=3	
J2	Discharged alive	Yes=1, No=0	
J3	In-hospital mortality	Yes=1, No=0	
J4	Composite poor outcome (regression variable)	ICU Admission and/or Death=1, Improved/Stable=0	
K. Eligibility checklist			
K1	Age ≥18 years	Yes=1, No=0	
K2	Acute stroke confirmed by CT/MRI	Yes=1, No=0	
K3	First admission	Yes=1, No=0	
K4	Arrived within 72 hours	Yes=1, No=0	
K5	TIA excluded	Yes=1, No=0	
K6	Traumatic ICH excluded	Yes=1, No=0	
K7	Stroke mimics excluded	Yes=1, No=0	
K8	Complete dataset available	Yes=1, No=0	
